# Enhanced recovery after surgery pathway reduces the length of hospital stay without additional complications in lumbar disc herniation treated by percutaneous endoscopic transforaminal discectomy

**DOI:** 10.1186/s13018-021-02606-z

**Published:** 2021-07-17

**Authors:** Wang Duojun, Zhang Hui, Lin Zaijun, Ge Yuxiang, Chen Haihong

**Affiliations:** 1 Department of Spine Surgery, Shidong Hospital, Yangpu District, Shidong Hospital Affiliated to University of Shanghai for Science and Technology, 999 Shiguang Road, Shanghai, 200438 People’s Republic of China; 2 Department of Orthopaedic Surgery, Minhang Hospital, Fudan University, 170 Xin Song Road, Shanghai, People’s Republic of China

**Keywords:** Enhanced recovery after surgery (ERAS), Lumbar disc herniation (LDH), Percutaneous endoscopic transforaminal discectomy (PETD), Length of hospital stay (LOS)

## Abstract

**Background:**

Enhanced recovery after surgery (ERAS) pathway in spine surgery is increasingly popular which can reduce the length of hospital stay (LOS). However, there are few studies on the safety and effectiveness of ERAS pathway in the treatment of single-level lumbar disc herniation (LDH) by percutaneous endoscopic transforaminal discectomy (PETD). The aim of this study was to investigate whether ERAS can reduce LOS of patients with single segment LDH treated by PETD.

**Methods:**

We reviewed the outcomes of all LDH patients (L4/5) who had been treated with PETD at our institution. Quasi-experimental study was adopted between patients treated in an ERAS after PETD with those rehabilitated on a traditional pathway. The two groups were analyzed for LOS, operation time, complications, visual analog scale (VAS), Oswestry Dysfunction Index (ODI), hospitalization expenses (HE), and improved MacNab efficacy assessment criteria (MacNab).

**Results:**

A total of 120 single segment LDH patients (ERAS pathway 60 cases, traditional care pathway 60 cases) who were selected from January 2019 to January 2021 met the inclusion criteria. There was a significant difference in mean LOS postoperative VAS scores and ODI on the 3rd day after surgery between the two groups (*P* < 0.05). The incidence of complications and HE were similar in the two groups (*P* > 0.05). The mean LOS decreased from 3.47 ± 1.14 days to 5.65 ± 1.39 days after application of ERAS pathway (*P* < 0.05).

**Conclusions:**

The ERAS pathway reduced LOS without resulting in additional complications after PETD. These findings support the application of the perioperative ERAS pathway in the treatment of single-level LDH with PETD.

**Level of evidence:**

Level IV, therapeutic

## Introduction

ERAS (enhanced recovery after surgery) is a multimodal clinical pathway designed to reduce surgical stress and postoperative catabolism and promote the recovery of postoperative body function [1, 2]. The core of this concept is to follow the principle of evidence-based medicine, integrate and optimize perioperative intervention measures on the basis of multidisciplinary cooperation, promote postoperative recovery, shorten LOS, reduce postoperative complications, and reduce the risk of readmission [1–3]. The concept originated in the late twentieth century cardiac surgery, first proposed by Professor Kehlet Denmark. Subsequent surgery in the colorectal and upper gastrointestinal tract has been shown to be safe and effective [4–7]. According to previous research, applying ERAS pathway to joint surgery can shorten LOS and improve satisfaction and safety after discharge [8]. In recent years, the ERAS model has been gradually extended to spinal surgery, but there are few reports on the application of ERAS model in spinal endoscopy, especially in PETD. The purpose of this study was to evaluate the clinical value of the ERAS clinical pathway in the perioperative management of lumbar PETD.

## Materials and methods

### Research design

This was a quasi-experimental design study that enrolled 120 patients with single-level lumbar disc herniation (L4/5) admitted between January 2019 and January 2021. We compared a prospective cohort (ERAS pathway: 60 cases) with a historical standardized care pathway cohort (traditional care pathway: 60 cases). Ethical approval was obtained from the internal review board, and orthopedic departmental approval was gained to proceed with prospective data collection. Inclusion criteria were as follows: (1) clear lumbar disc herniation, MRI, and CT examinations have shown L4/5 segment disc herniation and (2) consistent with surgical indications, contraindications to surgery have been ruled out. Written and oral informed consent was obtained from each patient prior to inclusion in the study. Exclusion criteria were as follows: (1) complicated with lumbar instability, lumbar deformity, lumbar spinal stenosis; (2) patients with severe basic medical diseases who cannot tolerate surgery; (3) previous history of cerebral hemorrhage, cerebral infarction, myocardial infarction, gastrointestinal bleeding, hepatic and renal insufficiency, chronic obstructive pulmonary disease, gastroduodenal ulcer, and lumbar surgery; and (4) patients who were unable to follow oral or written instructions.

All patients underwent surgery in our hospital, and each patient was reasonably evaluated and enrolled. Age, sex, duration of onset, LOS, operative time, complications, VAS, ODI, and MacNab were compared between the two groups. The primary study outcome was LOS, and secondary outcomes were ODI, MacNab, operative time, complications, HE, and VAS scores.

### Orthopedic ERAS clinical pathway

The basic components of the multidisciplinary and multimodal ERAS approach we used are shown in Table 1. Preoperative health education includes not only traditional education (including admission education, dietary guidance, telling patients to stop smoking and alcohol, aspirin and other drugs, introducing safety precautions, giving psychological nursing, and instructing deep breathing and cough exercises), but also detailed oral and written education on diseases and surgical techniques. The operating room nurse introduces the operating room environment and operating position. The skin condition and vascular condition of the patients were evaluated. Psychiatrists can provide detailed personalized and professional psychological counseling and use anti-anxiety and depression drugs when necessary to improve the preoperative mental state of patients. Midazolam was sedated 1 h before surgery at 0.07-0.08 mg/kg (IM), and age over 60 was reduced to 0.05-0.06mg/kg. An oral dose of 400 mg celecoxib was reduced to 200 mg on the day of surgery in elderly patients (> 70 years old) or patients with a low BMI (< 25 kg/m^2^). One percent lidocaine was used for local anesthesia, with a maximum amount of 300 mg. Four-milligram ondansetron hydrochloride (IV) was given at the same time as anesthesia to prevent intraoperative gastrointestinal reactions. The intraoperative anesthesiologist monitored the vital signs. According to the intraoperative situation, it was necessary to give 40-mg parecoxib sodium (IV). Use insulation blanket, heating fan, etc., to maintain the temperature is not lower than 36 °C. Postoperative fluid replacement is not required, and early high-quality diet is encouraged. Nutritionists assessed the nutritional status of patients and make a perioperative nutrition plan, and rehabilitation physicians make postoperative lumbar and back muscles and core muscles. Postoperative multi-mode analgesia, advanced analgesia, active drug administration, and reduction of opioid use were adopted, so as to reduce the incidence of respiratory system, gastrointestinal tract, and other related complications, and then achieved the purpose of early postoperative adaptive training.

**Table 1:** Perioperative management of two groups of patients with lumbar disc herniation

Implementation projects	ERAS group	Traditional group
**Preoperative**		
Sedation and analgesia	Yes	No
Education	Pre-operative education program + pre-operative counseling	Traditional education
Skin preparation	Area skin cleansing + operation area skin pre-depletion	Routine operations
Diet	No need to fast water or water deprivation	fasting 6 h, water deprivation 2 h
Prevention of gastrointestinal reactions	Application of serotonin receptor antagonists	No
**Intraoperative**		
Anesthesia monitoring	Anesthesiologist for intraoperative testing, if necessary, analgesia and other drugs	No
Prevention of hypothermia	Keep warm and prevent low temperature	No
Operation	Local subcutaneous injection of ropivacaine to relieve postoperative incision pain	No
**Postoperative**		
Postoperative analgesia	Active administration, avoiding opioid use, NSAIDs use	Analgesia on demand
Postoperative rehydration	No	Intravenous injection of dexamethasone 10 mg/day, for a total of 3 days
Postoperative nutrition	Nutritionists develop perioperative nutrition plans	Normal diet
Exercise of lumbar back muscle function	The rehabilitation physician formulates the rehabilitation exercise plan of psoas dorsal muscle and core muscle group after operation	Exercise of lumbar back muscle function
Getting out of bed	3 h postoperative	48 h postoperative

*ERAS* enhanced recovery after surgery

### Surgery

**Fig. 1:**
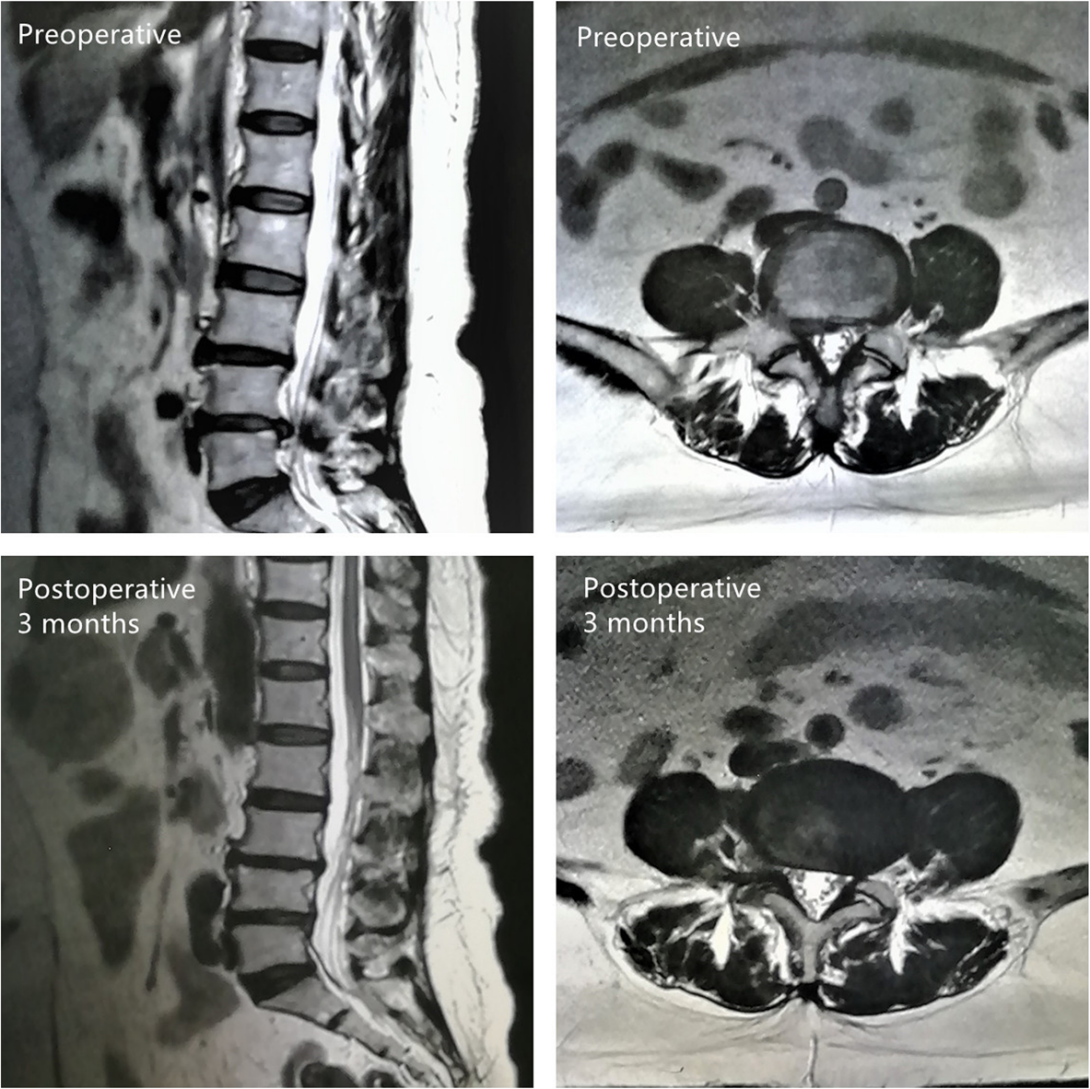
A case of a female patient, 58 years old, with low back pain and right lower limb pain, numbness and discomfort for 1 year, diagnosed with L4/5 degenerative huge lumbar disc herniation, and who underwent PETD treatment

Take the prone position, with the abdomen suspended, and the upper limbs in a comfortable position on the side of the head. Routinely sterilized drapes, first calibrate the midline of the spinous process and the parallel line passing through the upper edge of the intervertebral disc under the C-arm x-ray machine frontal fluoroscopy, and calibrate a lateral line passing through the posterior upper edge of the lower vertebral body to the upper articular process under lateral fluoroscopy. The intersection of this line and the parallel line of the upper edge of the intervertebral disc is the puncture point or 11-14 cm next to the L4/5 segment. For patients with obesity or intervertebral foraminal stenosis, the lateral distance should be increased accordingly. Ten milligrams per milliliter lidocaine was used for local infiltration anesthesia of the skin and soft tissues of the puncture path, and the TESSYS spinal foraminal endoscopy system was used for puncture. The puncture direction with 18-G puncture needle is 15–20 with the horizontal plane. The camber angle was punctured to the anterior and lower edge of the upper articular process of the lower vertebral body, and 0.5% lidocaine was injected around the articular process of 2 to 3 ml. A 22G puncture needle was inserted into the intervertebral disc through the 18-G puncture needle lumen, and the intervertebral disc was contrasted with a contrast agent. Remove the 22-G puncture needle, insert the guide wire through the 18-G puncture needle cavity, remove the puncture needle, take a 7-8-mm incision on the skin with the guide wire as the center, apply different types of soft tissue expansion catheters to gradually expand the surgical channel, and bite out with a serrated reamer The anterolateral part of the upper articular process of the lower vertebral body is bone, which enlarges the intervertebral foramen. Finally, a 7-mm diameter working sleeve is placed, and an intervertebral foramina is placed through the working sleeve. Different types of nucleus pulposus and nucleus scissors are used to remove the herniated disc tissue for decompression and go deep into the intervertebral disc space to remove the loose nucleus pulposus tissue, explore and loosen nerve roots, use radiofrequency ablation to assist hemostasis and shrink the ruptured annulus. During the operation, monitor the improvement of the patient’s symptoms in real time. If symptoms such as dural sac pulsation and nerve root relaxation are seen during the operation, it indicates that the decompression is sufficient. There was a case shown in Fig. 1. The patient, female, 58 years old, with low back pain and right lower limb pain, numbness, and discomfort for 1 year, was diagnosed with L4/5 degenerative huge lumbar disc herniation and underwent PETD treatment.

### Statistical methods

Statistical Package for Social Sciences (SPSS) (version 24.0, IBM, Armonk, NY, USA) Statistical software was used for data analysis. Measurement data were expressed as mean ± standard deviation (^−^x ± s). Two independent samples *t* test was used for comparison between the two groups. Enumeration data were presented as cases or rates. Chi-square test or Fisher’s exact probability method was used. *P* < 0.05 was considered statistically significant.

## Results

From January 2019 to January 2021, 120 patients with lumbar disc herniation (ERAS pathway, 60; traditional pathway, 60) were respectively selected to follow the ERAS pathway or traditional pathway. There were no statistically significant differences in age, sex, and course of disease among groups (Table 2). Local anesthesia was used as the main anesthesia method in both ERAS group and traditional group. The mean operative time in the ERAS group was lower than that in the traditional group, but it was not statistically significant.

**Table 2:** Basic characteristics of patients in two groups

	Traditional group (*n* = 60)	ERAS group (*n* = 60)	*p* value (*t*/*χ*^2^)
Age	48.60 ± 5.80	47.92 ± 5.89	0.525 (0.637)
Gender (female/male)	31/29	28/32	0.584 (0.300)
Course/month	13.82 ± 5.64	13.73 + 6.01	0.933 (0.085)
BMI	24.5 ± 4.35	25.2 ± 3.83	0.351 (0.936)
Average operating time (minutes)	70.75 ± 9.63	67.55 ± 10.84	0.090 (1.710)

Our results showed that the median LOS in the ERAS group was 3–4 days (average, 3.47 ± 1.14 days) compared with 5-6 days (average, 5.65 ± 1.39 days) in the traditional group (Table 3). The difference in mean LOS between the two groups was statistically significant. In addition, there was a significant increase in the number of patients in the ERAS group who left the hospital within 3 days of surgery.

**Table 3:** Average length of stay, visual analog score (VAS), ODI index (%), and MacNab

	Traditional group (*n* = 60)			ERAS group (*n* = 60)			*p* value (*t*/*Z*)
LOS	5.65 ± 1.39			3.47 ± 1.14			*P* < 0.0001 (9.393)
Preoperative VAS scores	7.25 ± 0.79			7.52 ± 1.17			0.141 (1.481)
VAS scores 1 day after operation	3.33 ± 0.60			2.25 ± 0.82			*P* < 0.0001 (8.233)
VAS scores 2 days after operation	3.07 ± 0.66			1.87 ± 0.50			*P* < 0.0001 (11.226)
VAS scores 3 days after operation	2.25 ± 0.47			1.47 ± 0.54			*P* < 0.0001 (8.440)
1 month postoperative VAS scores	1.13 ± 0.50			1.0 ± 0.45			0.137 (1.497)
Preoperative ODI	62.65 ± 14.62			61.45 ± 12.51			0.630 (0.483)
ODI 3 days after operation	19.55 ± 7.24			16.35 ± 6.80			0.014 (2.496)
ODI 1 month after operation	6.65 ± 2.15			6.07 ± 3.16			0.242 (1.176)
Evaluation criteria of improved MacNab efficacy	Excellent	Good	Fair	Excellent	Good	Fair	0.583 (0.545)
	50	5	5	52	5	3	
Hospitalization expenses (Ұ)	32,193.4 ± 4883.46			31,234.45 ± 4314.52			0.257 (1.140)

The clinical results of the two groups are shown in Table 3. There was no statistical difference in VAS scores between the two groups before operation. Compared with the traditional group, patients in the ERAS group had significantly lower VAS scores for 3 consecutive days after surgery. There was no significant difference in VAS scores between the two groups at 1 month postoperatively.

All patients in the ERAS group achieved early exercise and got out of bed 3 h after surgery. This was in contrast to the control group, which showed only patients moving out of bed 48 h after their surgery. Patients with early exercise of ERAS were discharged from hospital successfully within a shorter period of time after surgery. The ODI scores of the ERAS group were significantly improved 3 days after surgery, but there was no statistically significant difference 1 month after surgery. In addition, there was no statistical difference in the MacNab, as shown in Table 3. The total cost of hospitalization in the ERAS group was lower, but it was not statistically significant. Although the hospital stay in the ERAS group was shorter, the perioperative period required more expenditure. In conclusion, we conclude that evidence-based ERAS pathways have clinical significance for postoperative rehabilitation of patients with lumbar disc herniation, especially during the perioperative period.

Postoperative complications are listed in Table 4 for the two groups. There were 6 complications in the ERAS group and 9 in the traditional group after operation. There was no statistical difference (Table 4) (1 month follow-up after operation).

**Table 4:** Comparison of postoperative complications between the two groups

	Traditional group (*n* = 60)	ERAS group (*n* = 60)	*p* value (*χ*^2^)
Complications	9	6	0.408 (0.686)
Cerebrospinal fluid leak	1	0	
Nerve damage	2	1	
Incision infection	0	1	
Deep infection	0	0	
Lumbar and leg pain	2	2	
Urinary tract infection	1	0	
Cerebrovascular accident	0	0	
Myocardial infarction	0	0	
Respiratory infections	1	1	
Gastrointestinal reactions	2	1	

## Discussion

The goal of ERAS is to optimize perioperative management based on evidence-based medicine to achieve rapid postoperative recovery [1–3]. Currently, more and more ERAS management strategies have been applied in the field of surgery, and remarkable clinical results have been achieved. However, there is no unified standard for the implementation of ERAS concept for PETD. The implementation of ERAS requires the cooperation of multiple departments, and it is difficult to carry out the routine operation in the hospital, which requires routine training and education for doctors and nurses [9–11]. The ERAS pathway was adopted to promote early movement of patients, reduce postoperative pain, shorten LOS, and maintain good early function [1, 2].

Lumbar disc herniation is common in middle-aged and elderly people, and the ERAS pathway can promote early movement of patients out of bed and reduce the risk of bed-related diseases. Using percutaneous foraminal discectomy to treat lumbar disc herniation, combined with perioperative ERAS pathway, can more highlight the clinical significance of minimally invasive treatment and help to improve patients’ confidence in treatment and clinical treatment satisfaction rate. Debono et al. applied the ERAS model to lumbar fusion surgery and found that the postoperative recovery time was significantly shortened, and the incidence of complications and hospitalization costs were significantly reduced [12]. Feng et al. applied the ERAS model to minimally invasive transforaminal lumbar fusion, and the ERAS group also showed a shorter surgical duration and hospital stay compared to patients not managed with ERAS, but a similar complication rate in both groups [13]. Our study found that using PETD in the treatment of single-level lumbar disc herniation not only promoted patients to get out of bed early and exercise rehabilitation training but also reduced postoperative pain and hospital stay.

### Psychological assessment and preoperative education

Patients with lumbar disc herniation often have psychological problems such as depression, anxiety, and depression due to long-term back and leg pain, and the fear of surgery also increases the psychological burden of patients [14]. Previous studies have shown that preoperative anxiety and depression are negatively correlated with postoperative functional recovery and quality of life [15]. Therefore, it is particularly important to pay attention to the patient’s mental state, preoperative psychological evaluation, and psychological intervention. In addition, some studies have found that detailed hospitalization and preoperative education for patients with spinal diseases before admission can effectively relieve the tension and anxiety of patients, reduce the surgical stress response of patients, shorten the LOS, improve patient satisfaction, and help patients achieve the purpose of rapid recovery [16, 17]. In this study, the patients of ERAS pathway were invited for consultation with psychiatrists after admission and preoperative comprehensive education mode to receive detailed personalized and professional psychological counseling, and anti-anxiety and depression drugs could be used if necessary to improve the preoperative mental state of patients and accelerate their recovery.

### Preoperative pain management

Perioperative preoperative pain management mainly advocates multi-mode analgesia, including preoperative advanced analgesia, intraoperative wound local anesthesia analgesia, and reasonable postoperative analgesia. Advanced analgesia is a preoperative analgesic intervention to prevent or reduce postoperative pain, reduce the amount of postoperative analgesics, and accelerate the recovery of patients. Postoperative local analgesia around the surgical incision can reduce the course of early postoperative pain, and the application dose of postoperative analgesics is significantly reduced [18]. Reasonable postoperative pain is one of the most important postoperative management contents in ERAS pathway. Postoperative pain will increase patients’ bed time, increase the risk of cardiovascular and cerebrovascular accidents, and reduce patients’ subjective desire for early rehabilitation exercise, which is the primary reason for prolonging the LOS of orthopedic patients [9, 19]. The traditional pathway is to take appropriate action when a patient complains of excruciating pain. The ERAS pathway is to give analgesia until the anesthetic is completely gone. Opioid drugs can delay the recovery time of gastrointestinal function; cause postoperative nausea, vomiting, and other gastrointestinal dysfunction; and prolong the LOS, so non-steroidal anti-inflammatory drugs are used for postoperative analgesia in the ERAS intervention mode [20]. In this study, preoperative consultation with anesthesiologists was invited to develop a perioperative pain management plan. Advanced analgesia and multimodal analgesia were used in ERAS group to effectively reduce pain. Anesthesiologists can also conduct multi-dosage and multi-target combined analgesia through intraoperative monitoring, combining with the patient’s pain situation, to further alleviate the early postoperative pain symptoms. The use of selective COX-2 inhibitors not only reduces gastrointestinal reactions but also reduces opioid doses and opioid-related oversedation [8]. In this study, multimodal analgesia was used in the ERAS group. Compared with the traditional group, the postoperative VAS scores were statistically significant. It is precisely because the ERAS pathway emphasizes the use of pain management and advanced analgesia that these adverse factors can reduce the impact on the body’s stress response and thus promote rapid recovery.

### Postoperative nutritional management and functional recovery

Before surgery, nutritionists develop perioperative nutrition programs for patients and encourage high-protein, high-fiber, and high-vitamin diets, which not only help protect gastrointestinal mucosa, and accelerate the recovery of organ functions [5]. Besides, reducing the amount of fluid input after surgery can ensure the early movement of patients out of bed, which is conducive to early postoperative functional rehabilitation and can shorten the length of postoperative hospital stay [21]. In this study, patients in the ERAS group did not receive postoperative fluid rehydration, so early intake of high-quality diet was encouraged to facilitate early functional exercise. The rehabilitation physician formulated the postoperative rehabilitation and exercise plan for the lumbar back muscle and core muscle group, and the patients could get out of bed with the waist circumference under the guidance of the medical staff 3 h after the operation to enhance the strength of the lumbar back muscle, promote blood circulation, and reduce the risk of venous congestion, deep vein thrombosis, and prolapse pneumonia and other complications. At the same time, early bed activity can accelerate the recovery of gastrointestinal peristalsis, promote appetite, improve body resistance, promote wound healing, and help patients return to normal work and life as soon as possible [4, 5]. The results of this study showed that the postoperative 3 days ODI score of ERAS group was lower compared with the traditional group.

There were still shortcomings in this study, mainly limited by the small number of cases and short follow-up period. In addition, we have not reported more detailed results, such as hospitalization costs, patient satisfaction, and the rate of reoperation after 1 month, which is a limitation of our current ERAS study. Moreover, we did not evaluate the impact of age, gender, and disease course on the implementation of ERAS strategy. To be more specific to determine which types of patients are truly beneficial, the follow-up needs to be further studied.

In conclusion, in the treatment of single-level LDH by PETD, the optimization of perioperative ERAS intervention mode through multidisciplinary collaboration can reduce postoperative LOS and achieve the purpose of rapid recovery without resulting in additional adverse events. This has not previously been reported. Further studies should be undertaken to determine the relative importance of various individual measures and the longer-term functional outcome in patients treated on ERAS.

## Data Availability

The datasets used and/or analyzed during the current study are available from the corresponding author on reasonable request.

## Abbreviations

ERAS: Enhanced recovery after surgery
LOS: Length of hospital stay
LDH: Lumbar disc herniation
PETD: Percutaneous endoscopic transforaminal discectomy
VAS: Visual analog scale
ODI: Oswestry Dysfunction Index
MacNab: Improved MacNab Efficacy Assessment Criteria
